# A nomogram model for predicting ocular GVHD following allo-HSCT based on risk factors

**DOI:** 10.1186/s12886-022-02745-9

**Published:** 2023-01-23

**Authors:** Wen-hui Wang, Li-li You, Ke-zhi Huang, Zi-jing Li, Yu-xin Hu, Si-min Gu, Yi-qing Li, Jian-hui Xiao

**Affiliations:** 1 Department of Ophthalmology, Sun Yat-Sen Memorial Hospital, Sun Yat-Sen University, 107 West Yanjiang Road, Guangzhou, 510120 China; 2 Department of Endocrinology, Sun Yat-Sen Memorial Hospital, Sun Yat-Sen University, Guangzhou, 510120 China; 3 Department of Hematology, Sun Yat-Sen Memorial Hospital, Sun Yat-Sen University, Guangzhou, 510120 China

**Keywords:** Allogeneic haematopoietic cell transplantation, Chronic ocular graft-versus-host disease, Nomogram, Prediction model, Ocular surface disease index (OSDI)

## Abstract

**Objective:**

To develop and validate a nomogram model for predicting chronic ocular graft-versus-host disease (coGVHD) in patients after allogenic haematopoietic stem cell transplantation (allo-HSCT).

**Methods:**

This study included 61 patients who survived at least 100 days after allo-HSCT. Risk factors for coGVHD were screened using LASSO regression, then the variables selected were subjected to logistic regression. Nomogram was established to further confirm the risk factors for coGVHD. Receiver operating characteristic (ROC) curves were constructed to assess the performance of the predictive model with the training and test sets. Odds ratios and 95% confidence intervals (95% CIs) were calculated by using logistic regression analysis.

**Results:**

Among the 61 patients, 38 were diagnosed with coGVHD. We selected five texture features: lymphocytes (LYM) (OR = 2.26), plasma thromboplastin antecedent (PTA) (OR = 1.19), CD3 + CD25 + cells (OR = 1.38), CD3 + HLA-DR + cells (OR = 0.95), and the ocular surface disease index (OSDI) (OR = 1.44). The areas under the ROC curve (AUCs) of the nomogram with the training and test sets were 0.979 (95% CI, 0.895–1.000) and 0.969 (95% CI, 0.846–1.000), respectively.And the Hosmer–Lemeshow test was nonsignificant with the training (*p* = 0.9949) and test sets (*p* = 0.9691).

**Conclusion:**

We constructed a nomogram that can assess the risk of coGVHD in patients after allo-HSCT and help minimize the irreversible loss of vision caused by the disease in high-risk populations.

**Supplementary Information:**

The online version contains supplementary material available at 10.1186/s12886-022-02745-9.

## Introduction

Allogeneic haematopoietic cell transplantation (allo-HSCT) can cure many patients with certain malignant and nonmalignant haematologic disorders [1]. However, it is associated with many acute and chronic complications, of which graft-versus-disease (GVHD) is one of the most frequent and severe, affecting the patient’s lifespan and quality of life [2]. Generally, according to the time of onset, GVHD is classified as acute (0–100 days after transplantation) or chronic (> 100 days after transplantation), but it is the characteristic clinical performance that determines whether the disease is chronic or acute,rather than the time of onset. And some clinical features could appear in both acute GVHD or chronic GVHD [3, 4].

As a systemic immune-related disease, GVHD can involve several organs [5]. Skin GVHD usually presents with erythematous or lichen planus-like manifestations. When it involves the gastrointestinal system, diarrhoea, nausea, and anorexia are common symptoms [6]. Lung GVHD is often characterized by bronchiolitis obliterans syndrome (BOS), which can severely impact the quality of breathing [7]. If the liver is involved, severe, potentially fatal liver function impairment can occur [8]. All the lesions produced by GVHD, regardless of organ involvement, could cause irreversible damage, threatening human life and health [5]. Chronic ocular graft-versus-host disease (coGVHD) usually involves the anterior segment of the eye, such as the cornea, conjunctiva, and meibomian gland, and typically manifests as dry eye disease (DED). CoGVHD usually occurs 2 years after transplantation with a morbidity of approximately 60% [9, 10] and potential impacts on patient quality of life. About 80% patients used tear substitutes to relieve eye disorders. About 60% patients are unable to work because of ocular and systemic GVHD [11]. Ocular GVHD (oGVHD) is closely associated with the presence of systemic GVHD [12]. However, the relationship between ocular disorders and liver function and other organ lesions is unclear.

At present, coGVHD is mainly diagnosed based on the presence of ocular manifestations, which usually require ophthalmologists with equipment, such as slit lamps, for professional evaluation. Patients with coGVHD at the early or slight stages may have few obvious symptoms; however, at these stages, the tear glands and ocular surface have already been damaged [13]. As the damage progresses, the patients can feel severe ocular discomfort and visual disorders, affecting quality of life. For patients with serious ocular abnormalities, effective treatment is poor and may place a huge financial burden on their families. Moreover, little research has been conducted on the use a model to predict chronic ocular graft-versus-host disease based on systemic risk factors. Therefore, the purpose of this study is to assess the probability of coGVHD according to routine variables (such as lung function tests, clinical biochemistry, routine blood tests, coagulation tests and others). We constructed and validated a nomogram model based on risk factors and vision-related quality of life scale scores to predict the probability of coGVHD. Nomogram models have been widely applied as highly accurate tools for predicting disease prognosis [14]. If the prognostic factor can be identified, regular ocular examination is definitely recommended at proper time. By using this model, unexpected ocular complications could be prevented after allo-HSCT. Additionally, our nomogram could offer some protective treatment for high-risk groups, even those who feel little ocular discomfort or are in an early disease stage.

## Methods

### Study design and population

This cross-sectional study included individuals who had undergone allo-HSCT presenting with ocular and systemic indicators. These patients visited the ophthalmology outpatient clinic at Sun Yat-sen Memorial Hospital, Sun Yat-sen University, from May 2018 to August 2021. The inclusion criteria were as follows: 1) late-stage disease, that is, > 100 days after allo-HSCT; 2) age between 16–60 years; and 3) professional ophthalmological examination. The exclusion criteria were as follows: 1) history of dry eye disease, corneal ulcers, unhealed keratitis and other anterior segment diseases unrelated to allo-HSCT; 2) ocular infection within 6 months; 3) pregnancy or elevated human chorionic gonadotropin levels; and 4) autoimmune diseases, such as Graves’ disease, scleroderma, Sjogren’s syndrome, or systemic lupus erythematosus. Finally, we enrolled 68 patients who had undergone allo-HSCT from their ophthalmology clinic visits. Of those, 6 had missing systemic data and 1 had undergone a secondary allogeneic transplantation and were excluded.

### Clinical indicators and evaluation

All subjects underwent allo-HSCT. When the patients presented to the ophthalmological examination, they completed the ocular surface disease index OSDI questionnaire, which is an effective 12-item questionnaire for assessing vision-related quality of life [15]. Then, basic data were collected from their medical records, including routine examinations for basic systemic conditions. Blood samples were collected after fasting. Subsequently, the samples were centrifuged at 3000 rpm for 10 min. Routine haematology-related variables were measured on a Sysmex XN-9000 analyser (Sysmex Corporation, Kobe, Japan). Routine biochemistry variables were obtained through a Beckman Coulter AU 5800 (Beckman Coulter, Brea, CA, USA). Coagulation function was obtained on a Sysmex CS-5100 system TM analyser (Siemens Healthcare Diagnostics, Erlangen, Germany). Immunological function was analysed on a Navios flow cytometer (Beckman Coulter, Brea, CA, USA).

At the time of each visit, we recorded their ocular conditions, including visual acuity, intraocular pressure, tear film break-up time, corneal fluorescein staining, and Schirmer’s test results. Their basic condition was also collected, including donor and recipient conditions, systematic GVHD, routine blood test results, liver function, coagulation function, and lymphocyte subpopulation analysis. The ocular conditions of patients after allo-HSCT were assessed by senior ophthalmologist according to the National Institutes of Health Consensus on Criteria for Clinical Trials in Chronic Graft-versus-Host Disease [16], combined with ocular surface damage and ocular medical treatments.

### Statistical analysis

Data were analysed in RStudio software. The R package mice and glmnet were employed in this study. All tests were two-sided, and a *p* value of < 0.05 was defined as statistically significant. For continuous variables, clinical data that were normally distributed are expressed as the mean and standard deviation, and the t test was used to detect the differences between two groups; clinical data that were nonnormally distributed are presented as the median and interquartile rage, and the Mann–Whitney U test was used to explore the differences between two groups. For categorical variables, data are expressed as the number of cases and percentages and were compared with the chi-square test and Fisher’s exact test. Odds ratios and 95% confidence intervals (CIs) for ocular GVHD were calculated by using univariate and multivariate logistic regression.

Before building the model, we organized the original data. We assumed that missing data in the research were missing completely at random and used multiple imputations to replace them [17]. Then, we conducted comparisons of data before and after multiple imputations. All statistical comparisons of these data were not statistically different, which can be seen in Supplementary Table 1. Imputations made no substantial difference to the model. Because of the rarity of coGVHD, in building and validating this model, we set all data as the training set, while the testing set consisted of 70% of the data selected at random. With the training set, to identify risk factors associated with ocular GVHD, we used least absolute shrinkage and selection operator (LASSO) regression, which minimizes excessive fitting or selection bias in basic features, and tenfold cross-validation to assess the model. After LASSO regression, logistic regression could be used to reduce factors that were highly correlated. We also constructed a nomogram to predict the morbidity of ocular disease. After constructing the model, we assessed it with the training set and validation set. To determine the efficiency and accuracy of the model, we used the receiver operating characteristic (ROC) curve and the area under the ROC curve (AUC), and the quality of the model was assessed with the Hosmer–Lemeshow test. Furthermore, decision curve analysis (DCA) was used to assess the clinical benefits of the nomogram.

## Results

### Subject characteristics

Among these 61 patients in our study, 38 were diagnosed with ocular GVHD according to the National Institutes of Health Consensus. The basic characteristics of the post–allo-HSCT patients in the dataset are shown in Table 1. Compared with the non-coGVHD patients, patients with coGVHD had potential hepatic and lung function abnormalities. They tended to have lower actual and predicted forced vital capacity (FVC), 1-s forced expiratory volume (FEV1) and percentage of total lung capacity in a single breath (TLC/SB%) and higher gamma-glutamyl transferase (GGT) (all *p* < 0.05). There was no significant difference between the two groups in terms of alanine transaminase, aspartate aminotransferase, or skin or gastrointestinal (GI) GVHD.

**Table 1:** Characteristics of non-coGVHD and coGVHD groups

Variables	Non-coGVHD (*n* = 23)	CoGVHD (*n* = 38)	*P*-value
age, years	36.30 (13.40)	38.40(10.80)	0.526
FVC, L	3.09 (0.87)	2.97 (0.92)	0.642
FVC act/pred, %	90.00 (15.00)	78.80 (19.50)	0.022
FEV1, L	2.65 (0.86)	2.45 (0.98)	0.437
FEV1 act/pred, %	95.60 [83.10;104.00]	83.10[61.00;92.00]	0.006
TLC-SB, L	4.83 (1.02)	4.80 (1.06)	0.928
TLC-SB act/pred, %	96.30 (15.30)	86.50 (12.40)	0.019
DLCO/VA ratio, mmol/min/kPa/L	1.19 (0.19)	1.33 (0.26)	0.023
DLCO/VA ratio act/pred, %	68.2 (11.8)	79.1 (14.7)	0.005
WBC, 10^9L	4.04 [3.38;5.20]	5.43 [4.47;7.25]	0.019
PLT, 10^9L	119 [86.5;173]	164 [111;222]	0.048
EOS%, %	1.70 [0.85;5.95]	1.65 [0.50;2.77]	0.168
ALT, U/L	30.0 [21.0;46.0]	36.0 [23.2;58.0]	0.489
AST, U/L	34.0 [30.5;49.5]	30.5 [25.0;47.5]	0.329
TBIL, μmol/L	12.9 [11.2;16.0]	11.9 [9.70;16.7]	0.602
GGT, U/L	29.0 [19.5;93.0]	112 [34.8;212]	0.003
ALB, g/L	41.7 (5.08)	38.4 (6.02)	0.025
LYM, 10^9L	1.38 (0.69)	2.44 (1.64)	0.003
CD3-CD19 + cells,%	7.60 [1.42;21.0]	13.5 [4.52;18.5]	0.362
CD3 + CD25 + cells,%	1.46 [1.18;3.19]	3.28 [2.90;6.04]	0.057
CD3 + HLA-DR + cells,%	73.2 (22.2)	57.5 (17.3)	0.021
CD3 + CD4 + CD25 + cells,%	1.67 [0.98;2.81]	3.49 [2.35;6.28]	0.015
CD3 + CD8 + CD25 + cells,%	0.04 [0.02;0.19]	0.18 [0.08;0.51]	0.105
IgG,g/L	11.5 (3.12)	12.1 (9.10)	0.713
IgM,g/L	1.11 (0.82)	1.10 (0.73)	0.966
IgE,IU/mL	36.0 [10.0;159]	20.0 [8.50;49.5]	0.134
PT,s	11.9 [11.4;12.3]	11.6 [10.8;12.2]	0.030
PTA,%	96.9 (10.9)	106 (18.3)	0.024
PT/R	1.04 (0.06)	1.00 (0.07)	0.033
PTINR	1.04 (0.06)	1.00 (0.08)	0.035
OSDI	2.28[0.00–5.556]	22.73[6.68–34.32]	< 0.001
Systemic QOL	77.4 (16.8)	70.0 (15.4)	0.094
Recipient sex,male,n%	9 (39.1%)	24 (63.2%)	0.119
Donor sex, male,n%	19 (82.6%)	24 (66.7%)	0.297
Acute GVHD,n%	12 (52.2%)	16 (42.1%)	0.617
GI GVHD,n%	4 (17.4%)	3 (7.89%)	0.409
skin GVHD	7 (30.4%)	19 (50.0%)	0.219

*FVC* Forced vital capacity, *act/pred* actual/predicted value, *FEV1* Forced expiratory volume in 1 s, *TLC-SB* Total lung capacity-single breath, *DLCO/VA* Diffusion capacity carbon monoxide per liter alveolar volume, *WBC* White blood cell count, *PLT* Platelet count, *EOS%* Eosinophilia%, *ALT* Alanine aminotransferase, *AST* Aspartate aminotransferase, *TBIL* Total bilirubin, *GGT* Gamma-glutamyl transpeptidase, *ALB* Albumin, *LYM* Lymphocyte, *PT* Prothrombin time, *PTA* Prothrombin time activity, *PT/R* Prothrombin time ratio, *PTINR* Prothrombin time International normalized ratio, *OSDI* Ocular surface disease index, *Systemic QOL* Systemic quality of life, *GI GVHD* Gastrointestinal graft-versus-host disease

### Feature selection and risk factor analysis for ocular GVHD

**Fig. 1:**
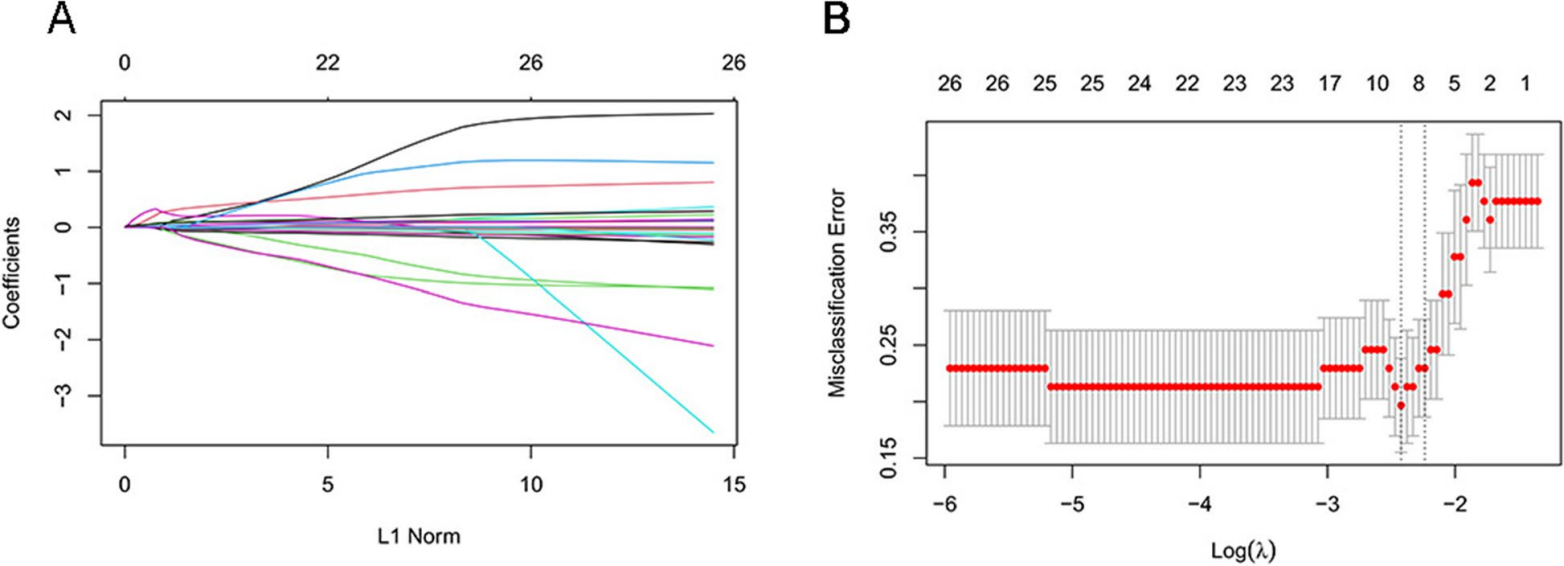
The least absolute shrinkage and selection operator (LASSO) for feature selection. **A** Model LASSO. Color lines represent the factors associated with ocular GVHD. The X-axis represents the alpha (cutoff), and the Y-axis represents the shrink effect value.LASSO coefficient profiles of the texture features. A coefficient profile plot was produced against the log (λ) sequence. Vertical line was drawn at the selected value via tenfold cross-validation, where optimal λ resulted in 7 nonzero coefficients. **B** the LASSO regularization parameter lambda was determined through tenfold cross-validation to select risks of ocular GVHD. The minimum criteria was used to draw dotted vertical lines at the optimal values. A λ value of 0.107 was chosen (1 standard deviation) according to tenfold cross-validation

We used LASSO regression to select important systemic ocular indices to accurately predict ocGVHD. After regression, we selected seven texture features: eosinophil %, lymphocytes, PTA, CD3 + CD25 + cells, CD3 + HLA-DR + cells, CD3 + CD8 + CD25 + cells, and the OSDI (Fig. 1A, B). Then, these seven texture features were screened by logistic regression. Five texture features were selected: lymphocytes, PTA, CD3 + CD25 + cells, CD3 + HLA-DR + cells, and the OSDI. As shown in Table 2, we also used multivariate logistic regression analysis to identify the OR values in the training and test sets.

**Table 2:** Risk factor analysis of coGVHD in development and validation datasets

Variables	Development dataset		Validation dataset	
	OR(95% CI)	*P* values	OR(95% CI)	*P* values
LYM,10^9/L	2.26(0.95–12.48)	0.241	0.73(1.54–7.36)	0.357
PTA,%	1.19(1.07–1.46)	0.014	1.07(1.20–1.53)	0.031
CD3 + CD25 + cells,%	1.38(0.95–2.24)	0.116	0.73(1.24–2.21)	0.404
CD3 + HLA-DR + cells,%	0.95(0.88–1.00)	0.111	0.88(0.96–1.02)	0.257
OSDI	1.44(1.18–2.11)	0.009	1.52(1.16–2.72)	0.047

### Construction and validation of the prediction model for ocular GVHD

**Fig. 2:**
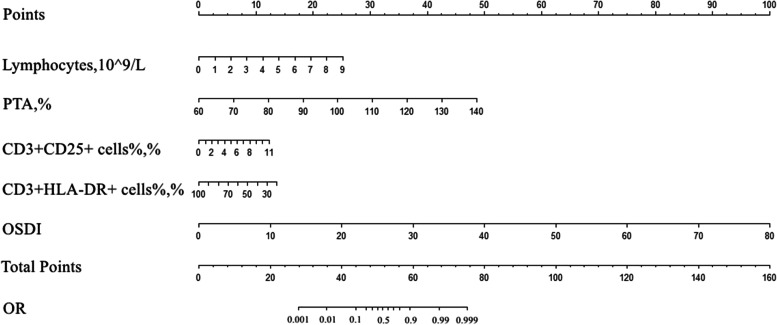
Nomogram for predicting morbidity of oGVHD. It is developed by the data of the training test

**Fig. 3:**
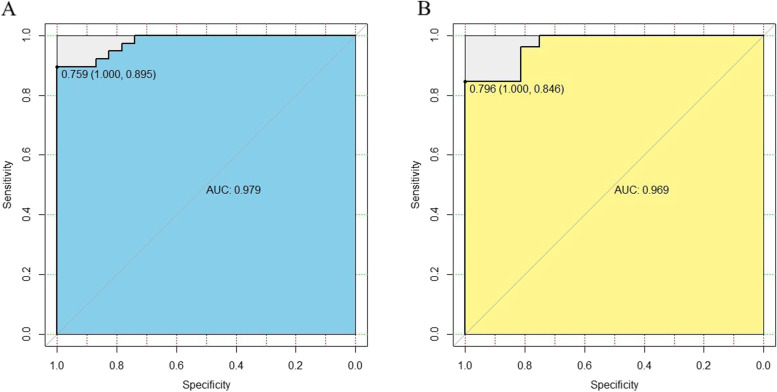
Receiver operating characteristic(ROC) curves to access the diagnostic efficiency of the nomogram in development and validation datasets. **A** ROC curve of the nomogram in the development dataset. **B** ROC curve of the nomogram in the validation dataset. AUC, area under the curve

To construct a more accurate predictive model, we constructed a nomogram based on the seven risk factors for oGVHD described above (Fig. 2). Then, we constructed the ROC curves to assess the predictive performance of the model with the training and test sets; the corresponding AUCs were 0.979 (95% CI, 0.895–1.000) and 0.969 (95% CI, 0.846–1.000), respectively (Fig. 3). The calibration curves of the nomogram showed great consistency between prediction and observation. The calibration curves of the nomogram was also conducted (Supplementary Fig. 1). The Hosmer–Lemeshow test was nonsignificant with the training (*p* = 0.9949) and test sets (*p* = 0.9691).

**Fig. 4:**
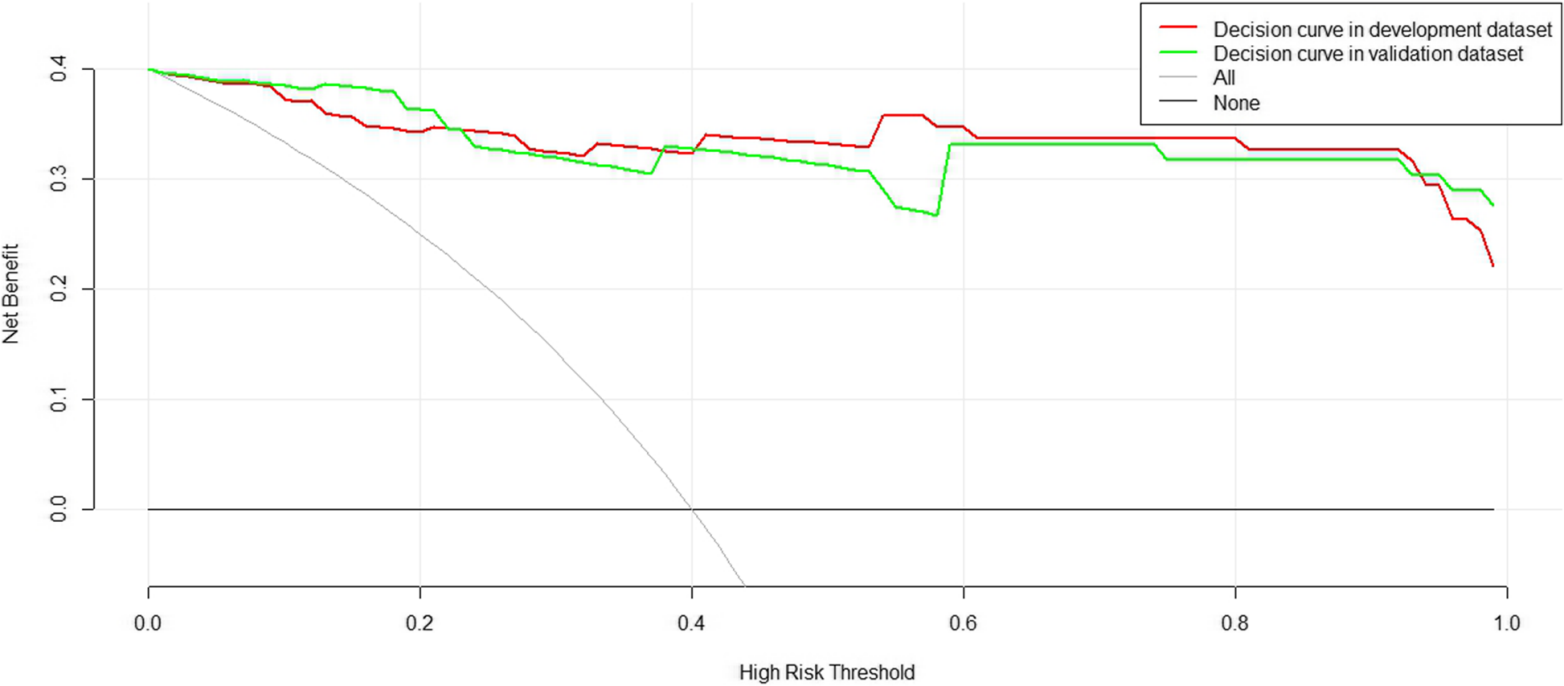
Decision curve analysis for the prediction model in the development (red color) and validation (green color) cohort. The y-axis represents the net benefit. Grey line represents all patients with oGVHD, black line represents no patients with oGVHD, green line represents decision curve in development dataset, red line represents decision curve in validation dataset

Furthermore, DCA was conducted to assess the reliability of the nomogram (Fig. 4). The resulting curve illustrates that the nomogram for ocular GVHD provides greater benefit than other schemes in both the training and test sets.

## Discussion

Patients may experience eye injury in the early stage of GVHD without any symptoms [18]. At this stage, ocular symptoms didn’t deteriorate. In the absence of conscious eye symptoms, it can be difficult for patients to take the initiative to visit the ophthalmology clinic, thus ignoring the early changes associated with the disease. Additionally, haematologists may focus more on diseases that seriously affect the lives of patients, such as those of the gastrointestinal tract, liver, and lungs, and neglect to remind their patients to visit the eye clinic. At present, only symptomatic treatment is available for patients whose severe eye diseases affects their survival and quality of life, but the effect is poor and results in inconveniences for the patient. By using this nomogram, the haematologist could calculate a score based on the relevant items in the patient's regular full-body review. If the patient has a high probability of eye disease according to the nomogram, a more professional evaluation is required even if the patient does not have any eye discomfort. For these patients or those at an early disease stage, relevant treatment or prevention can be given when necessary.

However, there is currently little research on the relationship between ocular and systemic GVHD, and the risk factors for ocular GVHD are still not fully understood. Dry eye disease is common in chronic ocular GVHD and has been seen in 60 to 90% of patients with systemic GVHD [19, 20]. Additionally, systemic GVHD was described as a risk factor for ocular GVHD [21]. However, few studies was able to determine the association between systemic and ocular GVHD. When the number of variables is large and much larger than the sample size, and there is serious multicollinearity between variables, LASSO regression can play its maximum utility. In our study, due to the large number of systemic conditions, so we chose this method to screen systemic data. We sought to construct a predictive model to determine the possibility of ocular disease according to the patient’s systemic condition.

The items we selected were chosen as predictors based on the strength of their univariate association with outcome through LASSO regression and logistic regression [22]. At last, the risk factors we found included lymphocytes, PTA, CD3 + CD25 + cells, CD3 + HLA-DR + cells, and the OSDI, as reflected in the comparison between groups, laying the foundation for the next step of constructing a predictive model. This nomogram was established using these seven risk factors and demonstrated a great diagnostic ability with both the training and validation sets. We hope our predictive model can be used widely for post allo-HSCT patients to predict the probability of coGVHD when they visit their haematologist.

Nomograms have been used in a variety of ocular diseases, such as for predicting glaucoma progression in patients showing disc haemorrhage based on risk factors [23] and in the prognosis of metastatic uveal melanoma according to the patient’s systemic condition, such as the percentage of liver involvement and lactate dehydrogenase (LDH) level [24]. Therefore, the concept of predicting a diagnosis of oGVHD according to the patient’s systemic condition through a nomogram is very promising. The results could be used to have the haematologist remind patients with early-stage ocular disease or those at high risk to visit the ophthalmology clinic for further assistance. Those in the high-risk group would particularly benefit, as such a visit may be helpful for developing a treatment or intervention plan.

We also aimed to explore systemic risk factors associated with coGVHD. The guidelines indicate that lung injury with GVHD is related to a decrease in FVC, which may indicate that the patient has bronchopneumonia obliterans, a condition known to be caused by GVHD [13]. However, there are few studies about the relationship between ocular injury and lung function. Here, we found that the actual and predicted FVC, FEV1 and TLC/SB% were related to ocular GVHD.

Liver damage is another main clinical manifestation of systemic GVHD [25, 26]. Studies have confirmed that changes in bilirubin and ALT are the most important manifestations of liver GVHD. Additionally, changes in the levels of bilirubin and/or ALT and GGT prior to the manifestation of the liver GVHD can be a sign of future liver GVHD in the guideline [27]. Interestingly, our research found that the liver index most closely related to ocular GVHD was GGT; this index has also been shown to be related to inflammatory disease and reactive oxygen species (ROS) production [28], and thus we speculate that changes in GGT may be related to liver inflammation in early liver GVHD.

In addition to those organs talked above, previous studies had indicated that ocular complications may be related with skin disease [29, 30]. Compared with patients without coGVHD, those with coGVHD had more skin lesions. The incidence of skin GVHD was higher in two groups, and the difference was not statistically significant. Therefore, we considered the reason why we don’t screen skin GVHD as risk factors might be associated with penalties.

Nomogram is widely used not only to predict ocular diseases, but also to predict other diseases, such as “Risk analysis of pulmonary metastasis of chondrosarcoma by establishing and validating a new clinical prediction model: a clinical study based on SEER database”, “Prediction of the risk of C5 palsy after posterior laminectomy and fusion with cervical myelopathy using a support vector machine: an analysis of 184 consecutive patients” and “Development and validation of a novel predictive model and web calculator for evaluating transfusion risk after spinal fusion for spinal tuberculosis: a retrospective cohort study” [31–33]. These models have good diagnostic efficiency, therefore, we believe that we should continue to promote the application of such models in ocular diseases.

This study aims to propose the use of systematic data to predict ocular GVHD to prompt patients to get a more professional assessment, but there are certain limitations. Although we used rigorous internal validation, this study lack additional validation with external data because of sample size. However, small sample is inevitable. Our present model provides and confirm a trend for this eye disease ceased by systemic disease. And a larger-scale multi-center study may be required, and the validity of the nomogram needed to be verified, too. In the next following studies, the sample size will expanded, and our model still need external validation. The index we choose is commonly used, and it was more relevant to the disease. It was also an indicator that was very suitable to be extended to other hospitals. Therefore, although the model had not been externally verified, it still had good test performance.

We believe that this method will have great diagnostic capacity and extensive application prospects. We hope to cooperate with other research groups and enroll more patients to refine and validate this model in the future. More dynamic observations of the ocular and systemic conditions will be given.Thus, our model will show enlarged applicability domain.

In summary, this proposes the use of systematic data to predict ocular GVHD so that the patient can be referred to an ophthalmologist to obtain a more professional assessment. We established a statistical model and demonstrated that it effectively predicts the progression of ocular GVHD caused by systemic disease.

## Supplementary Information

**Additional file 1: Supplementary Table 1.** Comparison between groups before and after data imputation.

**Additional file 2: Supplementary Figure 1.** Calibration curve to illustrate the calibration ability of the prediction model in development and validation datasets. A) Calibration curve to illustrate the calibration ability of the prediction model in development dataset. B) Calibration curve to illustrate the calibration ability of the prediction model in validation dataset.

## Data Availability

The datasets used and analyzed during the current study are available from the corresponding author on reasonable request.
