# Supplementary material for: A nomogram model for predicting ocular GVHD following allo-HSCT based on risk factors
Source: BMC Ophthalmol. 2023 Jan 23;23:28. doi: 10.1186/s12886-022-02745-9 (PMC9869507; doi:10.1186/s12886-022-02745-9)
Supplement: Supplementary file 1 — Additional file 1: Supplementary Table 1. Comparison between groups before and after data imputation. [file 12886_2022_2745_MOESM1_ESM.docx]

Supplementary Table 1 Comparison between groups before and after data imputation

| Variables | Original data(N=61) | After data imputation (N=61) | p-value |
| --- | --- | --- | --- |
| Group | 1.05 (0.99) | 1.05 (0.99) | 1.000 |
| Sex | 1.46 (0.50) | 1.46 (0.50) | 1.000 |
| Age | 37.6 (11.8) | 37.6 (11.8) | 1.000 |
| FVC | 3.02 (0.89) | 3.02 (0.98) | 0.989 |
| FVC act/pred | 83.2 (18.6) | 81.2 (20.5) | 0.572 |
| FEV1 | 2.53 (0.93) | 2.44 (1.01) | 0.624 |
| FEV1 act/pred | 88.9 [68.7;95.7] | 89.5 [68.7;101] | 0.641 |
| FVC/FEV1 | 82.4 (13.6) | 80.6 (15.4) | 0.512 |
| FVC/FEV1 act/pred | 97.7 (16.7) | 97.5 (18.4) | 0.959 |
| TLC/SB | 4.81 (1.03) | 4.99 (1.14) | 0.391 |
| TLC/SB act/pred | 90.4 (14.3) | 88.7 (15.1) | 0.550 |
| RV | 2.05 (0.76) | 2.09 (0.95) | 0.787 |
| RV act/pred | 125 (30.3) | 125 (34.2) | 0.921 |
| RV/TLC | 41.6 (8.08) | 42.5 (9.12) | 0.599 |
| RV/TLC act/pred | 138 (26.8) | 139 (28.9) | 0.714 |
| DLCO-SB | 5.98 (1.72) | 6.10 (1.94) | 0.724 |
| DLCO-SB act/pred | 66.0 (15.0) | 65.7 (16.2) | 0.926 |
| DLCO/VA | 1.27 (0.24) | 1.28 (0.26) | 0.872 |
| DLCO/VA act/pred | 75.0 (14.6) | 74.5 (16.8) | 0.844 |
| DLCO/VA act/pred | 4.76 [3.83;6.73] | 4.76 [3.83;6.73] | 1.000 |
| WBC | 3.83 (0.97) | 3.83 (0.97) | 1.000 |
| RBC | 117 (23.0) | 117 (23.0) | 1.000 |
| Hemoglobin | 154 [99.0;208] | 154 [99.0;208] | 1.000 |
| PLT | 48.0 (17.2) | 48.0 (17.2) | 1.000 |
| Neutrophils% | 8.93 (2.98) | 8.93 (2.98) | 1.000 |
| Monocytes% | 1.70 [0.50;3.00] | 1.70 [0.50;3.00] | 1.000 |
| Eosinophils% | 0.40 [0.20;0.70] | 0.40 [0.20;0.70] | 1.000 |
| Basophils% | 2.71 (1.91) | 2.71 (1.91) | 1.000 |
| Neutrophils | 0.49 (0.29) | 0.49 (0.29) | 1.000 |
| Monocytes | 0.15 (0.19) | 0.15 (0.19) | 1.000 |
| Eosinophils | 0.03 (0.06) | 0.03 (0.06) | 1.000 |
| ALT | 34.0 [22.0;56.0] | 34.0 [22.0;56.0] | 1.000 |
| AST | 33.0 [25.0;48.0] | 33.0 [25.0;48.0] | 1.000 |
| TBIL | 12.1 [9.90;16.1] | 12.1 [9.90;16.1] | 1.000 |
| GGT | 51.0 [27.0;164] | 51.0 [27.0;164] | 1.000 |
| ALP | 92.0 [74.0;133] | 92.0 [74.0;133] | 1.000 |
| Urea | 4.84 (1.69) | 4.79 (1.73) | 0.864 |
| Creatinine | 81.5 (32.9) | 83.0 (34.5) | 0.813 |
| Urea-to-creatinine ratio | 16.3 (5.20) | 16.2 (5.59) | 0.953 |
| Uric acid | 345 [267;442] | 345 [251;487] | 0.982 |
| Total protein | 67.1 (7.53) | 67.1 (7.53) | 1.000 |
| Albumin | 39.7 (5.87) | 39.7 (5.87) | 1.000 |
| Globulin | 27.4 (5.59) | 27.4 (5.59) | 1.000 |
| Albumin/Globulin | 1.52 (0.40) | 1.52 (0.40) | 1.000 |
| Total bile acid | 3.37 [2.40;7.40] | 3.20 [1.70;7.90] | 0.444 |
| CPK | 52.0 [28.0;77.0] | 53.0 [26.0;88.0] | 0.959 |
| LDH | 207 [164;248] | 208 [164;251] | 0.896 |
| CK-MB | 11.0 [9.00;15.0] | 13.0 [8.00;23.0] | 0.451 |
| HsCRP | 1.74 [0.76;4.30] | 1.79 [0.55;13.0] | 0.896 |
| ChE | 6872 [5485;8507] | 6712 [5463;8528] | 0.909 |
| LAP | 42.5 [33.5;74.9] | 46.1 [31.7;111] | 0.854 |
| Adenosine dehydrogenase | 15.2 (6.12) | 15.5 (7.35) | 0.827 |
| SOD | 154 (26.2) | 154 (28.8) | 0.985 |
| Free fatty acids | 519 [311;747] | 578 [301;931] | 0.352 |
| Lymphocytes | 1.98 (1.41) | 2.00 (1.62) | 0.942 |
| T cells | 72.5 (12.8) | 73.2 (13.0) | 0.773 |
| B cells | 12.8 [1.50;20.2] | 12.8 [1.45;22.4] | 0.879 |
| NK cells | 12.8 [7.58;17.8] | 12.3 [6.30;18.0] | 0.767 |
| CD4+ cells | 18.1 (8.11) | 18.6 (9.10) | 0.744 |
| CD8+ cells | 48.8 (15.8) | 48.6 (17.0) | 0.953 |
| CD4+CD8+ cells | 0.37 [0.20;0.66] | 0.38 [0.20;0.74] | 0.697 |
| CD3+CD25+ cells | 2.90 [1.31;4.76] | 3.28 [1.40;6.98] | 0.184 |
| CD3+CD38+ cells | 27.0 [13.8;52.7] | 18.9 [11.3;52.7] | 0.177 |
| CD3+HLA- cells | 66.0 (21.4) | 62.7 (25.2) | 0.495 |
| CD3+CD4+CD25+ cells | 2.47 [1.27;4.46] | 2.21 [0.94;5.21] | 0.631 |
| CD3+CD4+CD38+ cells | 5.51 (3.32) | 5.86 (4.13) | 0.642 |
| CD3+CD8+CD25+ cells | 0.10 [0.02;0.25] | 0.10 [0.01;0.52] | 0.722 |
| CD3+CD8+CD38+ cells | 20.3 [8.86;38.4] | 26.0 [7.01;53.3] | 0.395 |
| CD3+CD8+HLA- cells | 46.6 [32.2;66.9] | 56.7 [31.3;73.9] | 0.337 |
| Regulatory NK cells | 8.71 [4.63;15.2] | 8.06 [2.48;17.1] | 0.730 |
| TK cells | 88.6 (7.90) | 87.8 (8.95) | 0.626 |
| IgA | 1.00 (0.70) | 1.06 (0.76) | 0.659 |
| IgG | 11.9 (7.41) | 12.8 (9.37) | 0.575 |
| IgM | 1.10 (0.76) | 1.17 (0.86) | 0.626 |
| C3 | 988 [848;1162] | 1050 [851;1180] | 0.653 |
| C4 | 230 [178;305] | 224 [168;303] | 0.676 |
| Light chain κ | 2.67 (1.78) | 2.66 (1.76) | 0.965 |
| Light chain λ | 1.50 (0.85) | 1.54 (0.87) | 0.765 |
| IgE | 24.0 [8.75;64.2] | 26.0 [8.00;75.0] | 0.885 |
| PT | 11.7 [11.0;12.3] | 11.7 [11.0;12.3] | 1.000 |
| PTA | 102 (16.4) | 102 (16.4) | 1.000 |
| PT/R | 1.01 (0.07) | 1.01 (0.07) | 1.000 |
| PTINR | 1.01 (0.07) | 1.01 (0.07) | 1.000 |
| Fbg | 3.06 (0.95) | 3.06 (0.95) | 1.000 |
| APTT | 27.2 (3.65) | 27.2 (3.65) | 1.000 |
| TT | 18.8 (1.26) | 18.8 (1.26) | 1.000 |
| OSDI | 16.9 (18.3) | 16.9 (18.3) | 1.000 |
| Systemic QOL | 72.8 (16.2) | 72.8 (16.2) | 1.000 |
| Donor sex |  |  | 1.000 |
| Male | 43 (72.9%) | 45 (73.8%) |  |
| Female | 16 (27.1%) | 16 (26.2%) |  |
| Acute GVHD |  |  | 1.000 |
| No | 33 (54.1%) | 33 (54.1%) |  |
| Yes | 28 (45.9%) | 28 (45.9%) |  |
| GI GVHD |  |  | 1.000 |
| No | 54 (88.5%) | 54 (88.5%) |  |
| Yes | 7 (11.5%) | 7 (11.5%) |  |
| skin GVHD |  |  | 1.000 |
| No | 35 (57.4%) | 35 (57.4%) |  |
| Yes | 26 (42.6%) | 26 (42.6%) |  |
