# Supplementary figures and images for: A nomogram model for predicting ocular GVHD following allo-HSCT based on risk factors
Source: BMC Ophthalmol. 2023 Jan 23;23:28. doi: 10.1186/s12886-022-02745-9 (PMC9869507; doi:10.1186/s12886-022-02745-9)

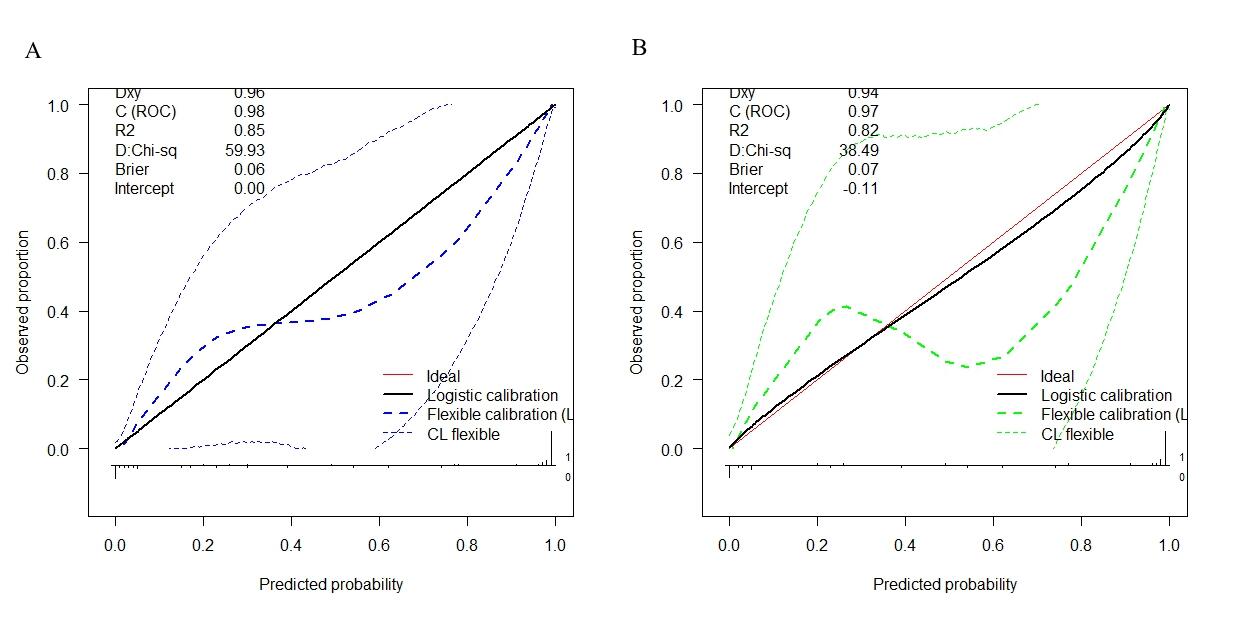

Supplement: Supplementary file 2 — Additional file 2: Supplementary Figure 1. Calibration curve to illustrate the calibration ability of the prediction model in development and validation datasets. A) Calibration curve to illustrate the calibration ability of the prediction model in development dataset. B) Calibration curve to illustrate the calibration ability of the prediction model in validation dataset. [file 12886_2022_2745_MOESM2_ESM.jpg]
